# Traumatic endophthalmitis following a crane pecking injury – An unusual mode

**DOI:** 10.3205/oc000038

**Published:** 2016-02-02

**Authors:** Prabu Baskaran, Seema Ramakrishnan, Pankaja Dhoble, Joseph Gubert

**Affiliations:** 1 Aravind Eye Hospital and Postgraduate Institute of Ophthalmology, Pondicherry, India

**Keywords:** traumatic endophthalmitis, crane pecking injury, beta-hemolytic streptococci

## Abstract

**Purpose:** To report a case of beta-hemolytic streptococcal endophthalmitis following crane-pecking injury.

**Case Report:** A twelve-year-old boy was brought to us by his father with history of crane beak injury in his right eye. On examination, his vision was 6/24 Snellen’s acuity. Anterior segment examination showed a full thickness two mm corneo-limbal tear at 1 o’clock with iris prolapse. Pupil showed peaking through the wound with a clear crystalline lens. There was no evidence of hypopyon in the anterior chamber and B-scan ultrasonography showed acoustically clear vitreous with an attached retina. Left eye was within normal limits. Primary corneo-limbal tear repair was performed within 24 hours from the time of presentation. Intra-operatively, the corneal surgeon noted turbid aqueous with minimal hypopyon. In view of clinical suspicion of infection, an intravitreal tap for culture was taken during the primary repair, and prophylactic intravitreal antibiotics were given. The culture report showed beta-hemolytic streptococci. Pars plana vitrectomy with intravitreal antibiotics was performed after 2 days as serial ultrasound scans showed appearance and worsening of endophthalmitis. A month after the surgery, his best corrected visual acuity improved to 6/12.

**Conclusion:** Ocular injuries resulting from bird pecking are very rare. We treated a case of full thickness corneo-limbal tear with endophthalmitis caused by beta-hemolytic streptococci following a crane-pecking injury. We recommend that injecting intravitreal antibiotics along with primary globe repair in case of severe/contaminated injuries and early pars plana core-vitrectomy would result in better outcome like in our case.

## Introduction

Ocular injuries due to bird pecking are very rare and not frequently reported in literature. Birds in general are considered to be harmless as they tend to fly away if one frightens them. But in rare instances, they can attack humans due to either territoriality or breeding [1], [2]. Children in particular are very susceptible to bird pecking due to their innate curiosity. We report a case of full thickness corneal tear with traumatic endophthalmitis caused by beta-hemolytic streptococci following crane-pecking injury. To the best of our knowledge, open globe injury with traumatic endophthalmitis following crane-pecking has not yet been reported in literature.

## Case description

**Figure 1:**
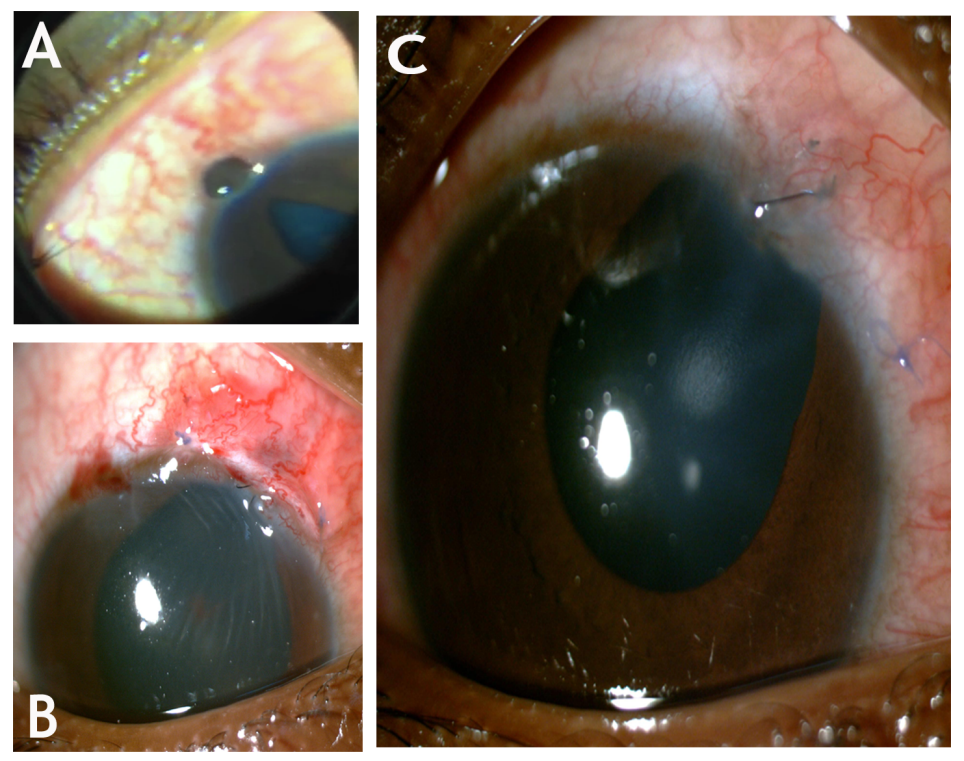
(A) Anterior segment photo taken using mobile camera and 20 dioptre condensing lens during indirect examination. The child was not co-operative for slit-lamp photography at the time of presentation. (B) Slit lamp picture showing dull glow due to vitreous exudates taken after corneal tear repair with iris abscission showing. Cornea shows folds on the descemet membrane due to tight sutures owing to the tissue loss. (C) Slit lamp picture showing quiet eye without any infection after core vitrectomy

**Figure 2:**
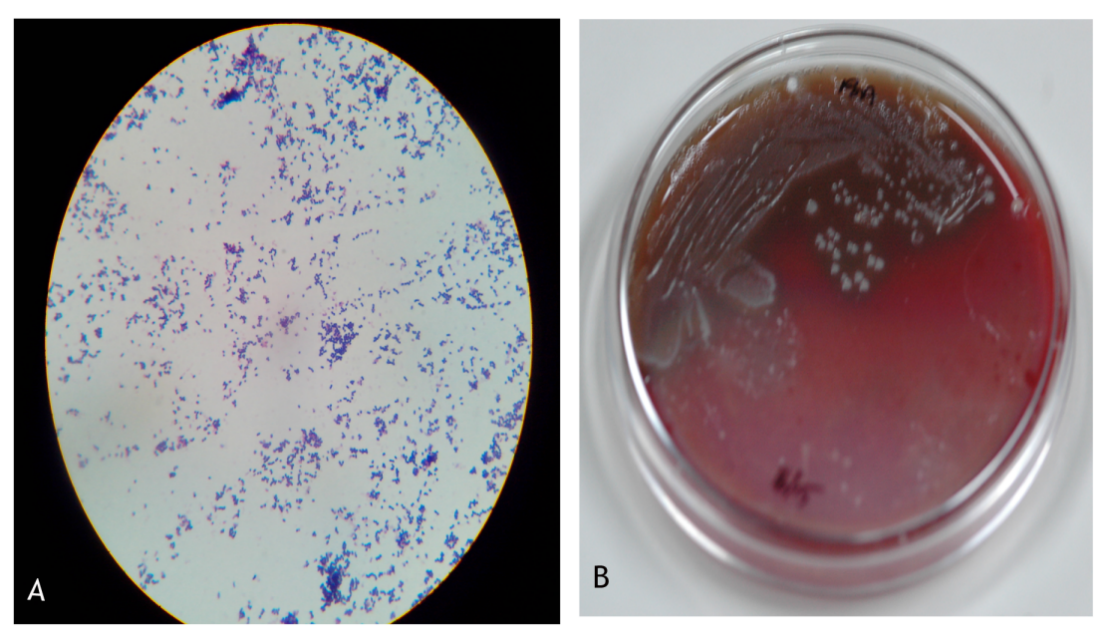
(A) Gram stain of the vitreous aspirate showing gram positive cocci in chains suggestive of streptococci. (B) Blood agar plate showing multiple colonies with complete hemolysis suggestive of beta-hemolytic streptococci

A 12-year-old boy was brought to our institute by his father in May 2015 with a week-old history of being pecked in the right eye by a crane. The bird was caged by his father, and the boy, out of curiosity, went close to it and the crane allegedly pecked on his right eye. On examination, his Snellen’s visual acuity was RE 6/24 and LE 6/6. Anterior segment showed a full thickness corneo-limbal tear 2 mm long at 1 o’clock with iris prolapse and a clear lens (Figure 1A (Fig. 1)). Though the patient was not co-operative for a detailed posterior segment evaluation, fundus glow was good. Gentle B-scan ultrasonography showed acoustically clear vitreous with attached retina. Left eye was within normal limits. Primary corneo-limbal tear repair was performed within 24 hours from the time of presentation (Figure 1B (Fig. 1)). Intra-operatively, the corneal surgeon noted turbid aqueous with minimal hypopyon. In view of clinical suspicion of infection, vitreous tap for microbiological evaluation was obtained along with the primary repair and prophylactic intravitreal vancomycin 1 mg in 0.1 ml and ceftazidime 2.25 mg in 0.1 ml were given. Gram staining of the vitreous aspirate showed evidence of gram-positive cocci in chains (Figure 2A (Fig. 2)) and multiple small, grayish white translucent colonies with variable zones of complete hemolysis on blood-agar suggestive of beta-hemolytic streptococci (Figure 2B (Fig. 2)). The colonies were found to be resistant to bacitracin suggesting a possible infection with *streptococcus agalactiae*. The exact species identification of beta hemolytic streptococci is quite tedious and requires multiple other tests unavailable with us. The organism was found sensitive to all antibiotics used for gram positive coverage. Serial ultrasound scans showed increase in vitreous echoes suggestive of exudates. We believe that the organisms must have entered the vitreous through the scleral side of the wound despite a clear lens. Vision in RE decreased from 6/24 (at the time of presentation) to 6/60. A three port pars plana core vitrectomy was done two days after the primary repair. Intravitreal vancomycin 1 mg in 0.1 ml and dexamethasone 0.4 mg in 0.1 ml were injected as per the sensitivity report. Intra-operatively, posterior pole was not involved with a healthy looking macula though the vitreous was full of thick white exudates. Following core vitrectomy, the eye became quiet and the patient was comfortable (Figure 1C (Fig. 1)). His best corrected Snellen’s visual acuity was 6/12 at one-month follow-up.

## Discussion

Injuries to the eye resulting from pecking of birds are uncommon and are most often caused by owls and roosters [1], [3]. The curved beak of these birds can result in serious ocular injuries including lenticular abscess and retinal detachment. Traumatic endophthalmitis occurs in 7% of penetrating injuries [4]. Roosters tend to attack at dusk and defend themselves from children by choosing the eye as a target of attack [1], [5]. Long-legged shore birds such as storks and cranes have been said to defend themselves against children by pecking exactly in the centre of the cornea, resulting in a triangular injury with prolapse of the uveal tissue [3], [6]. The white crane (*Egretta intermedia*) is a long legged shore bird with white body, long neck, dome shaped head and thick yellow beak with which it stalks its prey from shallow waters. It generally eats fish, frogs, crustaceans etc. The behavior of the bird can be aggressive in captivity. Management of these cases poses multiple challenges to the surgeon. Primary repair of the corneo-limbal tear can be very tricky as bird beak injuries are complex and can result in tissue loss. In such cases, the surgeon has to decide on placing a patch graft as suturing alone might result in severe astigmatism and poor wound apposition. Endophthalmitis is again very challenging in the pediatric age group due to various reasons like delay in diagnosis due to need for evaluation under general anaesthesia (EUGA), poor safety profile of systemic fluoroquinolones as they can cause cartilage damage and difficulties in vitrectomy due to stronger adherence at vitreo retinal interface. Dirty wound, delay in primary repair and ruptured lens capsule are independently associated with the development of post-traumatic endophthalmitis [1]. Essex et al. recommend the consideration of prophylactic intravitreal antibiotics if two of the above three risk factors are present [1], [4]. Though our patient presented early with an intact lens capsule, prophylactic intravitreal antibiotics were given along with the primary repair in view of the contaminated nature of the wound caused by the crane and also due to the presence of hypopyon in the anterior chamber at the time of primary repair. In conclusion, beta-hemolytic streptococcal endophthalmitis can occur due to pecking injury by a crane. We strongly believe in giving prophylactic intravitreal antibiotics along with primary repair of the wound whenever there is a high risk of infection. Also we recommend early core vitrectomy in bird beak injuries as delay might have deleterious effect on the final structural and functional outcome.

## Notes

### Competing interests

The authors declare that they have no competing interests.
